# ASMF: Ambient social media forensics chain of custody with an intelligent digital investigation process using federated learning

**DOI:** 10.1016/j.heliyon.2023.e23254

**Published:** 2023-12-03

**Authors:** Abdullah Ayub Khan, Xuzhuo Zhang, Fahima Hajjej, Jing Yang, Chin Soon Ku, Lip Yee Por

**Affiliations:** a Department of Computer Science and Information Technology, Benazir Bhutto Shaheed University Lyari, Karachi 75660, Sindh, Pakistan; b Department of Computer Science, Sindh Madressatul Islam University, Karachi 74000, Sindh, Pakistan; c Department of Computer System and Technology, Faculty of Computer Science and Information Technology, Universiti Malaya, 50603 Kuala Lumpur, Malaysia; d Department of Information Systems, College of Computer and Information Sciences, Princess Nourah bint Abdulrahman University, P.O.Box 84428, Riyadh 11671, Saudi Arabia; e Department of Computer Science, Universiti Tunku Abdul Rahman, Kampar 31900, Malaysia

**Keywords:** Ambient intelligence, Social media forensics, Blockchain technology, Internet of things (IoT), Federated learning, Digital investigation

## Abstract

Ambient Intelligence is a concept that relates to a new paradigm of pervasive computing and has the objective of automating responses from the system to humans without any human intervention. In social media forensics, gathering, analyzing, storing, and validating relevant evidence for investigation in a heterogeneous environment is still questionable. There is no hierarchy for automation, even though standardization and secure processes from data collection to validation have not yet been discussed. This poses serious issues for the current investigation procedures and future evidence chain of custody management. This paper contributes threefold. First, it proposes a framework using a blockchain network with a dual chain of data transmission for privacy protection, such as on-chain and off-chain. Second, a protocol is designed to detect and separate local and global cyber threats and undermine multiple federated principles to personalize search space broadly. Third, this study manages personalized updates by means of optimizing backtracking parameters and automating replacements, which directly affects the reduction of negative influence on the social networking environment in terms of imbalanced and distributed data issues. This proposed framework enhances stability in digital investigation. In addition, the simulation uses an extensive social media dataset in different cyberspaces with a variety of cyber threats to investigate. The proposed work outperformed as compared to traditional single-level personalized search and other state-of-the-art schemes.

## 1 Introduction

Media adoption has drastically increased over the past two decades, reaching almost the mark of more than 4.5 billion users, according to the report of Data-Re-Portal [1]. With the availability of a large number of data points, social media has been considered one of the major sources of information checks and background knowledge for smart collection; this intelligent gathering creates support for digital investigation, such as analysis of criminal activities, civil matters, compliance and regulatory matters, and issuance-related matters. These categories have been involved in the domain investigation of cyberbullying and defamation [2]. However, social media platforms consider social media a volatile source of data generation that is dynamic in nature. For instance, social media posts, including photos, memes, comments, and other types of information, can be uploaded and disappear in a matter of seconds or nullify individual chances that can be used for probatory reasons. Undoubtedly, various websites and different kinds of online services are available that record heuristic data on the Internet [3,4]. But there is no standard procedure, and most online services neglect social media platforms due to the extreme volatility of nature.

The traditional hierarchy of forensic investigations is to store all of their digital records at a certain point in time [3,4]. Preserving data from centralized storage in a data center provided by social media providers poses various challenging problems, such as required search warrants, technical problems related to cloud-based distributions, and cross-border jurisdictional data privacy and security. In the recent note, screenshots of any social media pages and posting that information on any platform are considered valid candidates for presenting in court [5]. However, it is most important that the preserved data of social media be treated with the same rigor as normal data collection from computer systems, ubiquitous devices, and centralized databases while conducting implicit investigations. The data stored is potentially used as a chain of evidence, which is one of the aspects of information litigation that were not foreseen at the initial level. So, for this reason, there is a requirement where forensics methodologies and related tools must be applied in situations to ensure stored media records maintain (i) source redundancy, (ii) manipulation, (iii) traceability, and (iv) discarding no volume for questioning when it is required for evidence presentation.

**Fig. 1:**
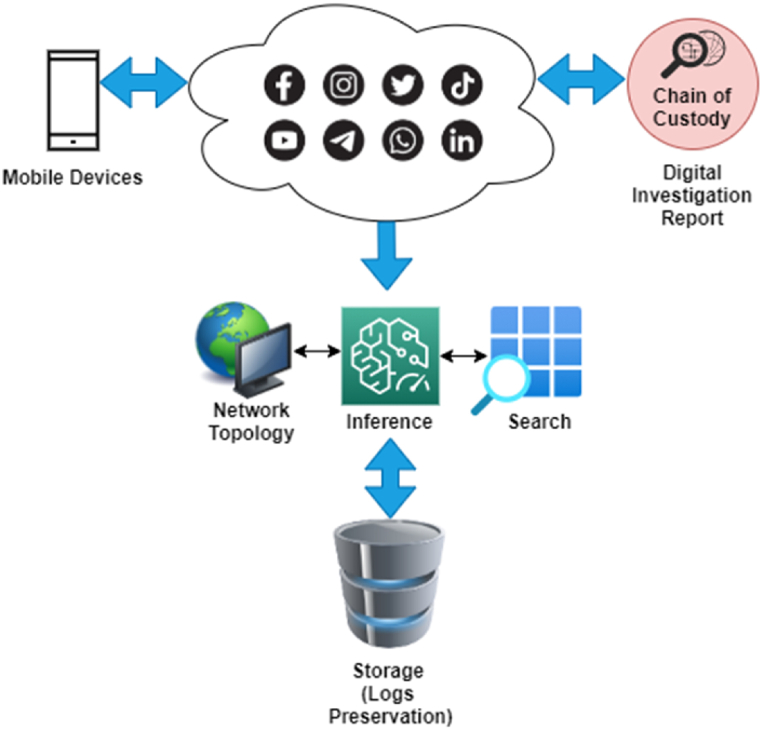
Recent hierarchy of digital investigation.

On the other hand, the chain of custody, preservation, and handling of this social media information collected from different platforms with different types of data formats is quite a complex task [3,5,6], as shown in Fig. 1. Moreover, the diverse nature of social media creates another perspective, such as the fact that there are no proper forensics capture and analysis state-of-the-art tools available; if they are found to be, then the software requires significant knowledge and expertise to run procedures. Even though a few social media forensics software programs are very fast at collecting and managing information from various platforms, they still lack the capabilities to resist duplication on social media, which contains a large amount of data and involves more human factors for management.

Recently, the concept of a collaborative investigational approach has been introduced, which means the integration of two technologies, such as social media analysis and digital forensics, in which Internet of Things (IoT)-enabled devices can be key to the detection of a closely related chain of evidence during the process of digital investigation in the cyber environment [5,6]. During the process of investigation, different techniques of digital forensics are used to uncover artifacts on ubiquitous devices, such as photos on smartphones, passwords, timestamps of locations, and third-party insights that require cross-analysis with the actual information. The existing technology assists digital investigators in different cases, including integrating, identifying data sources, and communication analysis, for the sake of building relationships in order to highlight spots and investigational parties. It is worth noting that integration enables robust decision-making capabilities along with providing a seamless environment to define paths for investigating chain-of-custody [[5], [6], [7]].

Undoubtedly, the role of digital forensics is critical in the social media environment. However, technological integration is becoming a powerful prospect for collecting relevant information in investigations [8]. The heterogeneous nature and disappearance feature of the social media platform pose a serious problem when executing parsing on the collected data and conducting examinations over the network. This issue requires a proper digital forensics methodology that requires accredited expertise to operate. Substantially, it is impossible to authenticate all the collected evidence generated in a row simultaneously. In order to make these prospects true, we need to make the system ambiently intelligent [8,9]. The introduction of ambient intelligence increases the role of correlation and strengthens findings without involving expert interference, which is inversely proportional to legal support addressing pertinent ethical and data privacy issues [9,10]. This new ambient-enabled social networking paradigm allows us to design and develop applications that connect other stakeholders in the immediate vicinity in a one-to-one channel, as compared to the current procedures.

Despite the concerns of standardized privacy and preservation, the technology could not be used as an indicator for future development based on the log-enabling functionalities [11]. However, most of the work in social media forensics is observational, which is related to multimedia. Its related detections, including manipulation, identification of media sources, and conditional results generated from the laboratory under different levels of investigation, pass through the knowledge of a forensics analyst. The research community of digital forensics recently pursued ambient-to-scale multimedia investigation for the purpose of analyzing real-world distributed applications (DApp) [12,13]. It is the purpose of this automation to automate the ecosystem for taking actions when receiving tampering, deceptive fake visual information, and interleaved operational activity sharing via an applicational environment. In addition, the extension of social media with multimedia forensics raises awareness of a few technological limitations, such as the processes of uploading, exchanging, and disappearing. Thus, we require a protected platform that can provide reliability in terms of information security under the general conditions of social media. In order to maintain this, a blockchain-Hyperledger-Sawtooth-enabled modular framework is designed for developing a secure DApp for the social media chain of custody and further investigational processes. In fact, during the process of development, the primary aim of the technology is to resist data alteration throughout; along with that, it reduces the cost of storage (because of the use of InterPlanetary File Storage (IPFS)) and network consumption (possible because of the consortium's distributed network structure) [13,14]. Although the current tools are able to detect patterns, they also hinder conventional mechanisms of digital forensics. In this scenario, the introduction of federated learning ensures posts are shared via a well-known social media environment and disappear from there, where it is worth noting that the changes cannot be involved in the signal in terms of data size and quality due to compression [[13], [14], [15]].

The major objectives of this research paper are discussed as follows:

• In this paper, we propose ‘ASMF’, a novel and secure framework using ambient intelligence with blockchain Hyperledger Sawtooth for forensic social media investigations.
• With the use of blockchain Hyperledger Sawtooth, a consortium distributed network design is proposed for the sake of managing both the transactions of digital investigation in the social media environment, such as implicit and explicit.
• A lightweight Proof-of-Stake (LPoS) consensus protocol is presented, along with the three different functions of the chain code to securely automate transactions.
• The concept of heterogeneous federated learning integrates with this proposed framework to tackle different social media resources from different platforms and filter them for further investigation.
• At the end of this paper, we critically review the other social media-related prospects and possibly address them along with the descriptive solution.

However, the rest of this research paper is structured, organized, and presented as follows: Section II discusses some of the critical challenges and limitations mentioned after reviewing previously published related research articles, such as social media and the role of ambient intelligence, federated learning, and blockchain distributed ledger technology. In Section III, a detailed description of the problem, the mathematical solution, and the possible outcome that is expected through this proposed work are discussed. The architecture named ‘ASMF’ is proposed to automate social media-based cyber forensics-enabled crime investigations, along with the details of the working hierarchy executions in Section IV. In Section V, the simulation results of the proposed ASMF are presented. Finally, we conclude this research paper in Section VI.

## 2 Related work

This section is divided into two categories that mainly highlight the level of work presented previously by the expert and the current gaps available. In addition, it also indicates technological integrations for the sake of futuristic maturity in social media forensics developments.

### 2.1 Social media forensics and ambient intelligence

The discussion of ambient intelligence in the social media environment is initiated after receiving a method of multi-agent-based simulations for testing and validation in ubiquitous computing domains [16]. The main purpose of this technology is to provide services that have some intelligence in order to maintain fast interactions with stakeholders. This motivation comes from the department of supply-chain management of a well-reputed multinational organization, where a large-scale system is unable to tackle a large number of stakeholders' queries that are associated with designed applications. It occurs due to the replacement of real tests that are impractical and creates an artificial society, which is highly required in order to facilitate humans [16,17]. Most of the recent developments in ambient social media are based on sufficiently descriptive analysis, which means that the ecosystem only allows requisite violations in the functionalities of the real-time system. A solution for UbiCom proposed previously mainly integrates small and cheap devices together, which are placed physically at one end and used virtually from another end in the environment of users [[16], [17], [18]]. This proposed solution is designed to analyze both the forensic examinations and the users’ simpler social simulations simultaneously.

Substantially, as per the report of IEEE Standard Glossary Software, data validation is a key prospect in the process of social media forensics chain of custody, which is defined as the hierarchy of examining an ecosystem or related components during the implementation process to identify whether the technology facilitates the requirement and verify [19]. On the other side, data verification integrates the current software examination with an individual aspect to determine whether the system satisfied the conditions under which the ecosystem starts management. However, in most cases, usability engineering methods failed to build an environment for testing, validating, and verifying [20]. In establishing a good digital investigation environment, a usability professional organization is required, along with some new paradigms that are collaborative in nature (such as Artificial Intelligence (AI), Augmented Intelligence, Machine learning, and the Internet of Things (IoT)) that help in the data collection testing, verification, validation, and debugging at the last stage before creating a chain of evidence in the social forensics environment [2,17,19,20].

### 2.2 Role of federated learning with blockchain in social media investigation

In the context of social media, a chronological chain-like structure of social networking is currently deployed, which is considered one of the most notable aspects of the centralized environment. Due to this, it leads to a large amount of mobile data traffic; on the other side, the demand for network consumption is also increased, such as bandwidth usage [21]. However, it is worth noting that the lack of discussion on the cost of computation for information processing affects the load of network distribution and organization, which is directly proportional to digital investigation. In order to maintain this, a self-organizing network is presented that aims to manage the complete pathway, which means it does not require details of source and destination nodes. Intercommunication between centralized networks can be carried out through encounters with the procedure of node-enabled block movement. In this process, two nodes shared data in such a way that it preserved the exchange to ensure stable communication among connected nodes. In addition, each node in social networks has a relatively better interconnection environment due to smooth interactions, which most probably affects the behavior in the social environment. The role of federated learning with security protocols is critical in the overall management of social media forensics investigations, as discussed in Table 1.

**Table 1:** Related works of social media forensics.

Proposed Methods	Research Highlights and Contributions	Limitations	Comparison with the Proposed ASMF Similarities/Differences
Machine learning, especially Support Vector Machine-enabled generative adversarial networks for learning edge-based computing attacks and out-poising [22]	• Proposed a novel poisoning attack detection mechanism for secure transactions in the social media environment. • A federated machine learning-enabled training dataset is designed, including a classifier for genuine data points.	• Underlying data distribution • Estimate bad optimization • Detect the area specifically, which is realistic for attacks	• Computational complexity • Compromising the classifier's use while utilizing machine learning algorithms • A bi-level optimization strategy is used
A distributed node-enabled block screening method using federated learning with the Internet of Things and blockchain in social networking platforms [23]	• In the social media environment, this paper investigates the protocol of node selection based on distributed proximal policy optimization. • Separates two processes: multi-threaded interactions and updates of the global network.	• Heterogeneous nodes and optimization problems • Cannot alleviate user privacy leakage • Interoperability issue	• Distributed near-end strategy in node selection • Markov decision process used • Distributed node-enabled community division mechanism
Network traffic anomaly detection using personalized federated machine learning [24]	• A concept of personalized federated anomaly detection for anomaly detection over a centralized network environment. • Tested on real network traffic and analysis.	• High false alarm rate • Use of self-coding of long- and short-term memory networks • Involvement of deep learning for fine tuning	• Data reconstruction-based detection methods • Prevent nonanthropogenic anomalous information interference
On the platform of wireless network communication, energy efficiency powered by federated machine learning [25]	• Q-V. Pham et al. proposed a novel joint algorithm for technological power control, transmission scheduling, efficiency, bandwidth management, computational resource organization, and energy fluctuations.	• Unavailable for terrestrial communications • Replace central server communication • Wireless powered communications	• Sustainable federated machine learning solutions • Interoperable solution • Joint algorithm used
With the capability of resource optimization in terms of communication over wireless Internet of Things networks using federated learning [26]	• This paper presents an infrastructure that aims to maximize the rate of information exchange over an IoT network using a LaGrange multiplier method. • Proposed a new paradigm or framework named CEFL, which aims to work on a subdomain of the given client scheduling and computational resource management-related problems.	• Scope of data security, privacy, and protected preservation issues • Jointly, communication optimization efficiency limitations	• Collaborative learning techniques in machine learning and edge networks • Linear search-enabled energy and network bandwidth allocations • Calculate communication efficiency as a priority task

## 3 Preliminary knowledge

In this context, we discuss basic knowledge with the notation related to the research problem that needs to be defined; along with that, we also formalize ambient intelligence and distributed automation-related issues as follows:

### 3.1 Problem description

**Fig. 2:**
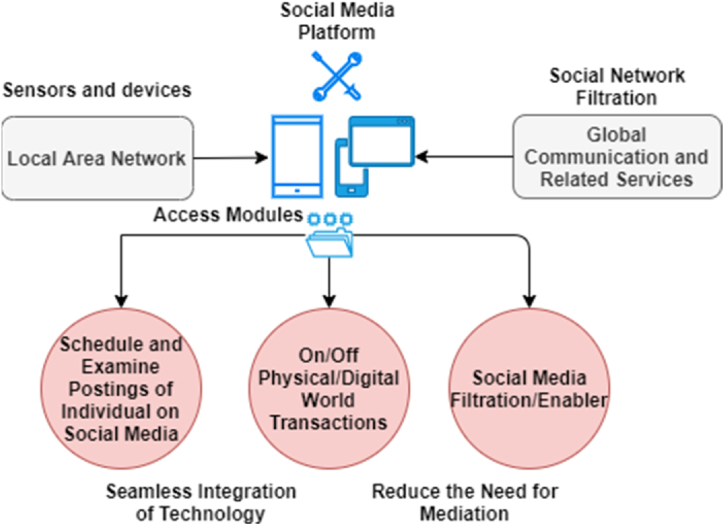
A pictorial description of the social media investigation hierarchy.

To enhance the way humans interact with internet technology to promote smooth and robust communication, reduce barriers to delays, and most importantly, enrich the standardized hierarchy. Social media is one such domain, but this paper extends to autonomous prospects in the digital forensics’ investigation environment for the sake of automated collection of unwanted occurrences of vulnerabilities that led to cybercrime. In this manner, ambient intelligence on social media platforms has been considered a good solution in recent times. This proposed technology not only involves improving the capabilities of close interactions in social media environments but also helps to capture logs in cyberspace. In order to achieve ambient intelligence in social media, the system depends on a large technological deployment, including Internet of Things (IoT)-enabled sensors and machine-to-machine interconnection via a distributed network. This proposed design increases the intelligence of the system that is used for decision-making but is unable to handle a large distribution of heterogeneous node requests, as shown in Fig. 2. Federated learning integrates with this system to manage multiple independent sessions, each aiming to capture, examine, analyze, optimize, preserve, and present its own occurrence verification and validation.

The problem of ambient intelligence with federated learning faced in social media forensics is addressed as follows:

• Embedded (E): Distributed social media platforms interconnection issues;
• Context awareness (CA): efficient detection and recognition of contextual situations posted on social media platforms;
• Adoptive hierarchy (AH): accuracy on change in response;
• Personalization (P): tailored in accordance with the user's needs and related problems;
• Anticipation (A): no standardized anticipation procedure while addressing the desires of the system's needs without conscious meditation.

### 3.2 Problem formulation and notations

In this paper, the proposed ASMF comprises four sub-portions: (i) blockchain Hyperledger Sawtooth, (ii) ambient intelligence, (iii) federated learning participation that schedules distributed nodes’ data, and (iv) with a single smart contract or chaincode and customized Proof of Stake (PoS) consensus protocols, as shown in Algorithm 1.

The notations and parameters of the ASMF are mentioned in Table 2. For Hyperledger Sawtooth, HS represents the blockchain-Hyperledger-Sawtooth-enabled distributed application (DApp) where the smart contract is deployed. It generates a string of Re-Encryption, Nu, and Tk, which denote the number of nodes that generate blocks (B_i_=(B_1_, B_2_, B_3_, …, B_n_)) in the designed chain created by the ASMF to identify transactions of chain-of-custody, while a pair of consortium channels serve as the implicit and explicit intercommunications throughout the process.

**Figure:**
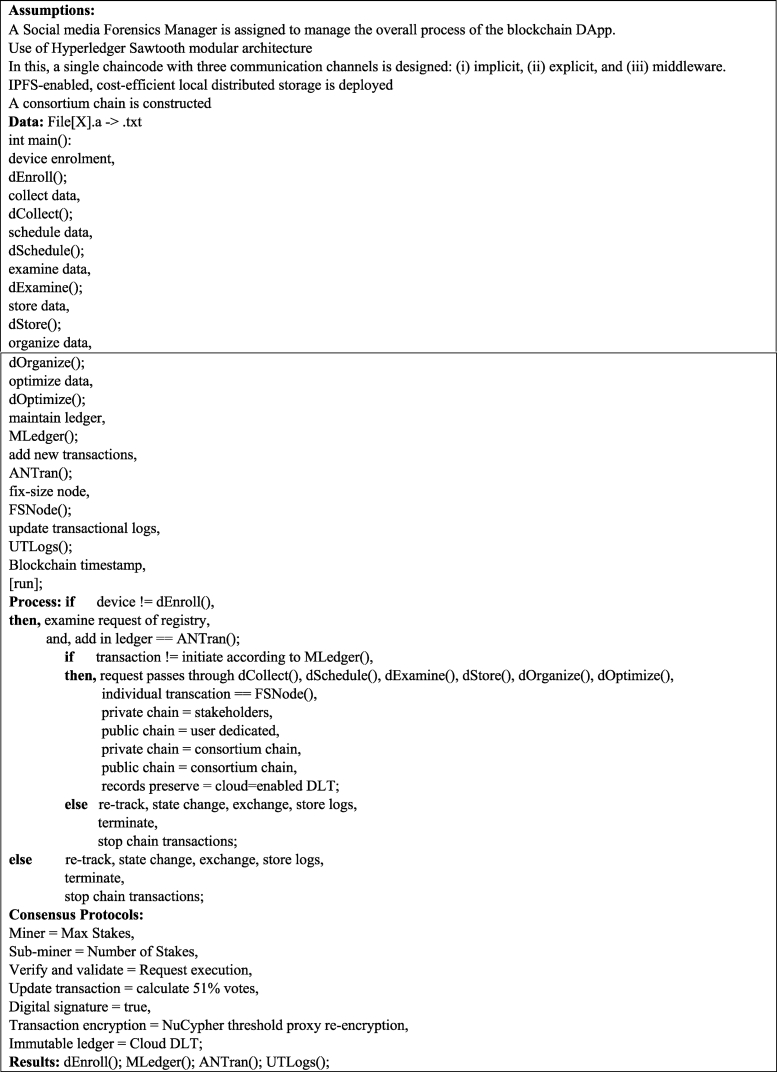

**Table 2:** Symbols of the proposed ASMF.

Symbols	Details
Nu	NuCypher Threshold Re-Encryption
S_i_	Number of stakeholders
DApp	Distributed application
Tk	Threshold key
R_i_	Random generation
H_i_	Heterogeneous nodes connectivity
B_i_	Nodes of blocks
r	Range of block size
P_i_	Proof generation
SMF	Social media forensics
M_i_	Distributed random number of mappings from r
G_i_	Gradient
PoS	Proof of Stake
HS	blockchain-Hyperledger-Sawtooth-enabled

Towards the privacy and security of the social media forensics ledger, the implementation of smart contracts using Linux-based Hyperledger technology with customized PoS protocols In this manner, verification and validation of individual transactions need to be verifiable randomly through heterogeneous federated learning (Hi), scheduling R_i_ = (R_1_, R_2_, R_3_, …, R_n_), and proof generation by enabling P_i_ = (P_1_, P_2_, P_3_, …, P_n_) and M_i_ = (M_1_, M_2_, M_3_, …, M_n_). This whole process is considered a public random function execution.

However, for the participating stakeholders, S_i_=(S_1_, S_2_, S_3_, …, S_n_) represents the number of participants of social media forensics investigators denoted as ith register via the ASMF DApp, uses Nu to generate its private random r generations, and the proof P_i_ = (P_1_, P_2_, P_3_, …, P_n_), which is becoming a seed to H_i_ = (H_1_, H_2_, H_3_, …, H_n_) that helps for the evaluation of the complete Gi of the processes.

Ambient intelligence collaborates with heterogeneous federated learning at the point of automation, while preserving the ledger and privacy prospects of participating stakeholders’ requests from being shared with others during the process of ASMF training is critical. Therefore, a blockchain-Hyperledger-Sawtooth-enabled modular infrastructure is used to run requests from stakeholders in a protected manner. It is possible because of the custom design of the PoS consensus protocol, where sensitive information from social media forensics is exchanged via consortium channels, which means both private and public transactions occur securely.

**Fig. 3:**
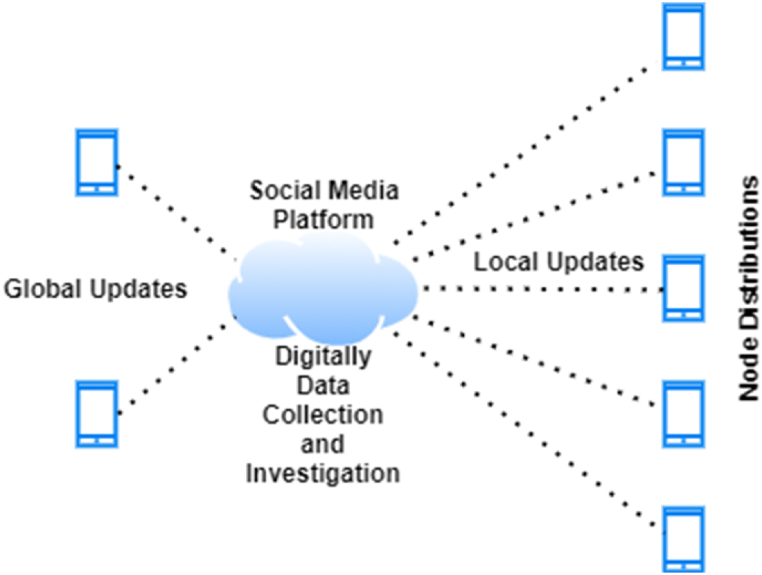
Hierarchy of federated processing.

The sub-objective of the proposed ASMF is to design a collaborative framework that securely preserves a social media forensics chain of evidence through a fair, verifiable process using heterogeneous federated learning, as shown in Fig. 3. Thus, the description of subdivisions is addressed as follows:

• Detection of unwanted social media activities;
• Standardized hierarchy;
• Digital investigation;
• Verification and validation;
• Privacy;
• Security;
• Preservation/chain of custody;
• Efficiency.

## 4 Proposed framework

**Fig. 4:**
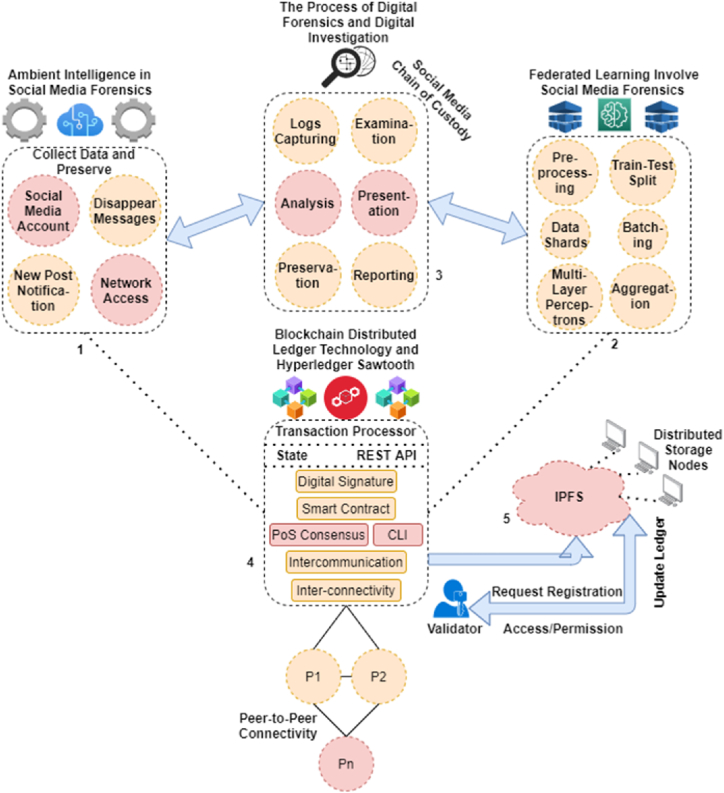
The proposed ASMF.

Fig. 4 presents a blockchain-Hyperledger-Sawtooth-enabled distributed ledger framework (named ASMF) for a novel and secure social media chain of custody and forensic digital investigations. In this proposed framework, the use of ambient intelligence with heterogeneous federated learning is critical. One is designed to manage the collection of social media data (such as posts) autonomously, and the other is to evaluate in terms of examining and analyzing these distributed records collected from different platforms of social media, such as Instagram, Snapchat, and Facebook. However, the major highlights of this proposed ASMF are discussed as follows:

### 4.1 Operations, executions, and flow controls

The proposed ASMF is created with the integration of five sub-domains of information technology, such as (1) Ambient Intelligence (AmI), (2) heterogeneous federated learning, (3) digital forensics, (4) blockchain Hyperledger Sawtooth, and (5) distributed storage.

The AmI is deployed in the social media environment for collecting data and preserving logs in the distributed storage autonomously. There is a need to analyze unwanted changes occurring throughout social media surfing. Also, to schedule investigations on social media posts to examine fake news or promotions, it analyzes the network logs in terms of IP addresses, from which we can determine where the post was uploaded from and disappeared on the platform. The components of AmI for critical investigations are as follows: (i) account login; (ii) logs of disappearing posts; (iii) altered posts as new; and (iv) network access. On the other end, the analysis of individual logs from the distributed platforms over the consortium network poses a serious issue. In this manner, the ASMF placed a middleware through the use of heterogeneous federated learning, whose main objective is to preprocess the collected evidence captured via the AmI, as shown in Fig. 4. Between these processes, digital forensics plays a vital role in the process of digital social media investigations. Each captured piece of data sent from the AmI to the heterogeneous federated learning domain is followed by the hierarchy of forensics investigation, such as examination, analysis, presentation, preservation, and reporting. No loss is required in the overall procedure. However, the prospect of heterogeneous federated learning is mentioned as follows:

• preprocessing, which means filtering and separation;
• train-test split;
• data shards;
• batching;
• multilayered perceptron, and
• aggregation.

### 4.2 Hyperledger Sawtooth-enabled chaincode design

With a few assumptions of ASMF design (as mentioned in Algorithm 1), we present a pseudo-implementation of the proposed smart contract/chaincode with four functions of DApp executions, such as dEnroll(), MLedger(), ANTran(), and UTLogs(). Initially, the ASMF starts with the dEnroll() function for the sake of verifying and validating requests for participation. The categories of stakeholders’ registration in the ASMF are as follows: (i) read and write; (ii) read; and (iii) read, write, and validate. However, the function ANTran() aims to record each log that occurs throughout the process of a transaction, from scheduling to delivery, including implicit and explicit communications. On the other side, MLedger() manages (such as organize, manage, and optimize) data in the distributed preservation structure and exchanges it among the participants of the chain, which is immutable in nature. In this paper, the Interplanetary File Storage (IPFS) System is used. The proposed ASMF and IPFS are capable of handling both on-chain and off-chain transactions in social media forensics. Finally, the function UTLogs() is designed to initiate the overall activities of the DApp, including request passes through dCollect(), dSchedule(), dExamine(), dStore(), dOrganize(), and dOptimize(). No redundancy or duplication in log preservation is allowed. In this manner, the customized PoS consensus protocol plays a crucial role in the scheduling of miner selection for those who have max stakes; moreover, the other activities of the designed PoS are as follows: (i) sub-miner = number of stakes; (ii) verify and validate = send request for execution; (iii) update/change transaction = calculate 51 % votes required; and (iv) digital signature = true value.

The main assumptions of this proposed DApp are that the social media forensics manager is the only person who counts as a validator other than the sub-validator of the ASMF; the use of the modular architecture of Hyperledger Sawtooth; the distribution of the architecture into three sub-domains (i) middleware, (ii) implicit channels, and (iii) explicit channels; and a consortium chain is constructed to tackle public and private transactions separately.

## 5 Results and discussions

Initially, this section mentions that the proposed work is simulated on three different platforms of social media, such as Instagram, Snapchat, and Facebook. In this manner, we use a benchmark dataset, which is the social media dataset from the Kaggle Instagram platform [[27], [28], [29], [30], [31], [32], [33]]. Overall, this data is based on more than 40 thousand pictures and almost a 3.3 GB large volume set.

The requirements of the system are highlighted as follows:

• Heterogeneous node connectivity;
• Intel Core i7 vPro chipset with 1 TB of internal storage and 32 GB of RAM;
• 10 Mbps local communication and 100 Mbps global communication.

**Fig. 5:**
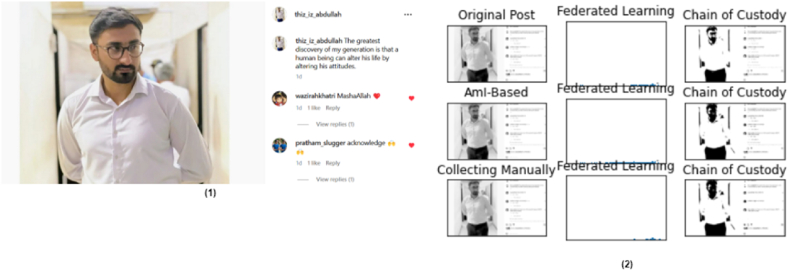
Social media post evaluation (1), where (1) the original uploaded post and (2) the results of the unwanted or altered post are obtained after the involvement of AmI, federates learning with digital forensics.

The simulation of ASMF is tested on a post uploaded on Instagram that disappeared or was deleted by the account manager within 30 min. The logs are acquired through the AmI, which is already recorded on the distributed ledger. Fig. 5 illustrates the relationship between the examination of the original social media post, the involvement of AmI for auto-log collections, distributed analysis using heterogeneous federated learning, data shards, batching, and creating a chain of custody that will be presented in court for further decisions.

**Fig. 6:**
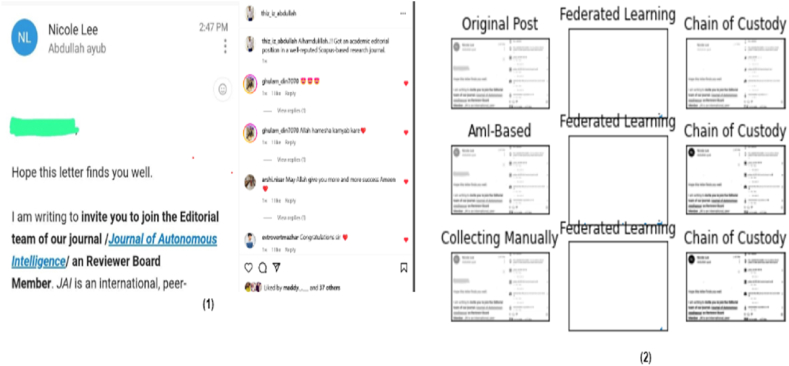
Social media post evaluation (2), where (1) the original uploaded post and (2) the results of the unwanted or altered post are obtained after the involvement of AmI, federated learning with digital forensics.

Whereas Fig. 6 shows the evaluation metrics whose constraints are original posts uploaded on social media platforms (such as Instagram and Snapchat), the role of AmI automation for logs is to collect them directly, perform distributed analysis using heterogeneous federated learning, perform train-test splits (as mentioned in Table 3), create data shards, batch (as discussed in Table 4), and create a chain of custody that will be presented in court for further decisions.

**Table 3:** Evaluation metrics (1).

Figure (s)	Train Score (%)	Test Score (%)	Batching	Overall %
Fig. 5	83.91 %	16.09 %	Batch normalization	91.10 %
Fig. 6	79.88 %	20.12 %	Batch normalization	89.63 %
Fig. 7	81.56 %	18.44 %	Batch normalization	89.89 %

**Table 4:** Evaluation metrics (2).

Pre-processing	Classifier	Analysis	Number of Shares	Overall Metrics%
Metadata extraction in Instagram	Post	Global	Single	96.22 %
Metadata extraction in Snapchat	Media files	Local	Multiple	91.22 %
Metadata extraction in Facebook	Post	Global	Multiple	93.22 %

However, the process of digital forensics helps in the examination of fake data uploaded on the platform, such as promotions or news, enabling the hierarchy of digital data detections and investigations. It includes individual aspects of data uploaded to the environment that pass-through collection, examination, analysis, presentation, preservation, and documentation, as shown in Fig. 4 and explained in Algorithm 1.

**Fig. 7:**
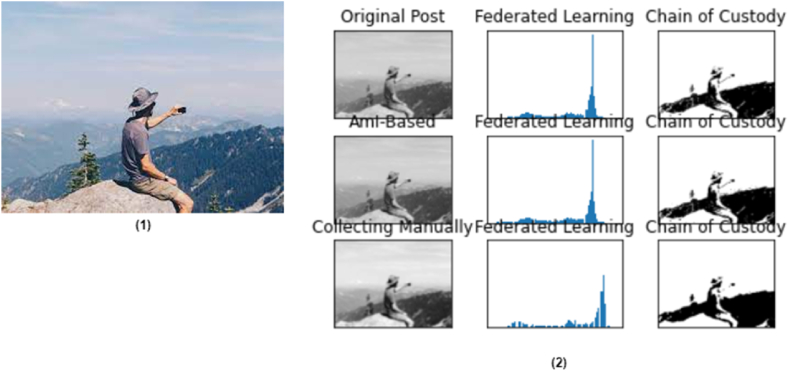
Social media post evaluation (3), where (1) the original uploaded post and (2) the results of the unwanted or altered post are obtained after the involvement of AmI, federates learning with digital forensics.

In the overall process, the role of heterogeneous federated learning is important to complete such a digital investigation procedure for the sake of creating an efficient social media chain of custody. A decentralized machine learning technique where a distributed middleware interactively maximizes the AmI parameters of logs of data collections without accessing individual features, as shown in Fig. 7. User heterogeneity is imposed, which is one of the crucial aspects of the proposed ASMF. Analysis of the drift of local and global network coverage, specifically. The details of the evaluation are mentioned in Table 4.

**Fig. 8:**
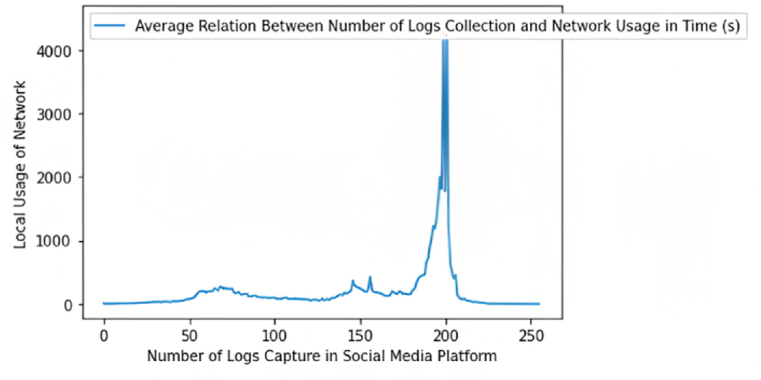
Relationship between the number of logs collected and the usage of the network over time (s).

The working hierarchy of the designed distributed network structure is illustrated in Fig. 8. In this evaluation, we examine the complete deliverance of logs collected through the AmI and the preservation of these data on the immutable ledger in what amount of time. The metrics of examination are the number of logs captured on the social media platform and the consumption of network bandwidth per cycle.

**Fig. 9:**
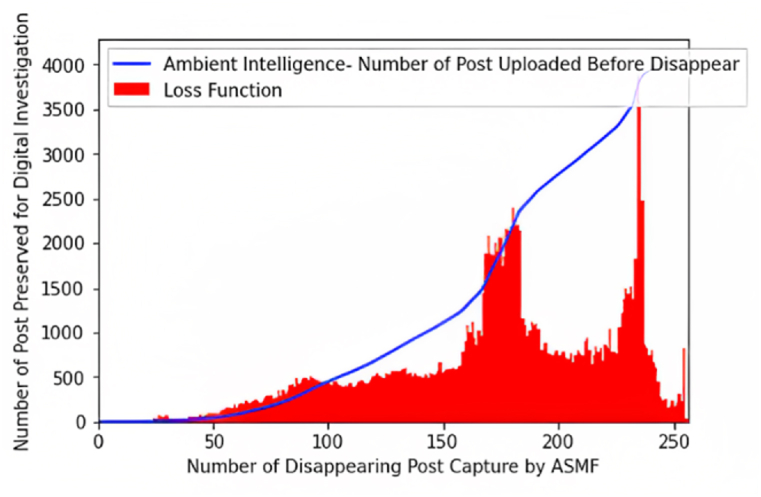
Evaluate the loss function while applying ambient intelligence.

Meanwhile, the proposed work simulates the cost of utilizing ambient intelligence. Fig. 9 shows the relationship between the number of posts uploaded and recorded autonomously. In this process, we analyze that the proposed ASMF lost (loss function) some values while preserving logs in IPFS.

In order to tackle this problem, we integrate a stop-and-listen distributed network mechanism that reduces the loss functions, whose metrics are the number of disappearing posts captured by ASMF and the number of posts preserved for further digital investigation, as shown in Fig. 9.

**Fig. 10:**
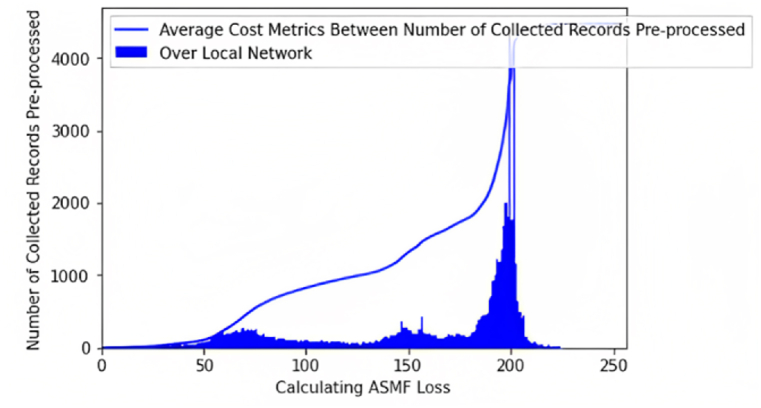
Average cost metrics between the number of collected records during pre-processing over the local network.

Fig. 10 illustrates the evaluation of the average cost consumed during preprocessing while capturing logs over local network connectivity. The metrics of the examination are calculated by calculating loss over the number of collections, as briefly mentioned in Table 4.

However, the evaluation matrices show the fluctuation towards betterment is balanced up to 7.39 %, such as in energy efficiency. And so, the automation accuracy increases up to 3.13 %, whereas the cost of computation and storage capability reduces to 5.34 % and 6.21 %, respectively.

We compared some of the state-of-the-art methods with the proposed ASMF; however, these methods are closely related (but not exactly) to the components of the proposed one: (1) Robust byzantine federated learning for DApp privacy, protection, and preservation; and (2) an autonomous lightweight authentication platform using blockchain and federated learning [27,28]. Undoubtedly, these works are presented in order to meet the problems of privacy and security in which a performance is concerned; along with that, there is a gap between the single use of technology, technique, and algorithm and the collaborative approach. In addition, these presented works only indicate the system's performance related to privacy and security but neglect the cost of technological consumptions such as computational energy, network bandwidth, and preservation. Substantially, Table 5 presents a comparative analysis in which we compare the proposed work with previously published articles. The evaluation metrics of the comparison are mentioned as follows (Table 5):

**Table 5:** Comparative analysis between the proposed ASMF and other state-of-the-art methods.

State-of-the-Art Method (1) [29,30]	State-of-the-Art Method (2) [31,32]	State-of-the-Art Method (3) [33]	Proposed Model
The metrics of comparative analysis are mentioned as follows::			
• Use of Blockchain DLT: Public Blockchain • Network structure: Permissionless network structure • Encryption method: hash-encryption (SHA-256) • Node size: Depends on the transactional data • Method for data management and optimization: Not applicable • Method for data transmission and delivery: N/A • Cost of log preservation: IPFS Blockchain • System's efficiency: N/A • System's Accuracy: N/A	• Use of Blockchain DLT: Public Blockchain • Network structure: Permissionless network structure • Encryption method: hash-encryption (SHA-256) • Node size: Not applicable • Method for data management and optimization: Deep neural nets • Method for data transmission and delivery: N/A • Cost of log preservation: IPFS Blockchain • System's efficiency: N/A • System's Accuracy: N/A	• Use of Blockchain DLT: Public Blockchain • Network structure: Permissionless network structure • Encryption method: hash-encryption (SHA-256) • Node size: Depends on the size of the transaction • Method for data management and optimization: Computational method • Method for data transmission and delivery: N/A • Cost of log preservation: IPFS Blockchain • System's efficiency: N/A • System's Accuracy: N/A	• Use of Blockchain DLT: Consortium Blockchain • Network structure: Hyperledger Sawtooth modular network architecture • Encryption method: NuCypher Proxy Re-Encryption Mechanism • Node size: 4 MB fixed size • Method for data management and optimization: Federated Learning • Method for data transmission and delivery: P2P consortium channel • Cost of log preservation: Cloud-enabled DLT is designed and deployed • System's efficiency: 3.13 % and 7.64 • System's Accuracy: 5.34 % and 6.21 %

### 5.1 Open research limitations

In this context, different open research problems are listed and analyzed, and a few of them are addressed with their possible solutions as follows:

#### 5.1.1 Scope of data privacy protection

Data privacy protection is a significant objective running throughout the process of remote sensing data; to address such concerns in real-time, proper use of blockchain DLT is required. Especially in smart cities, the personal information of connected nodes needs to be protected when joining or before the designed consortium chains, including individual node details, scheduling and processing hierarchy, satisfying each type of data recorded, and preservation management [34,35]. In this regard, a cloud-enabling local distributed ledger structure is integrated with blockchain immutable infrastructure and a NuCypher Threshold Proxy Re-Encryption mechanism to prevent malicious records and preserve all those records in a secure and cost-efficient manner. In addition, the tri-communication channels separate the implicit, explicit, and middleware resource transactions. It helps to examine the list of scheduled transactions effectively and efficiently. However, the explicit transactions from interoperable chains create a challenging problem, such as a lack of privacy during information exchange among them. To protect against this type of issue, NuCypher Re-Encryption with customized PoS consensus is used to integrate the current infrastructure of blockchain technology for land surface applications in smart cities [35,36].

#### 5.1.2 Outsource computation and remote sensing data

In the recent environment, cloud or fog computing technology is considered a mature concept because it offers storage scalability and computing power in a pay-per-use model. Receiving data from the sensing devices required computation, whereas the current scenario uses cloud or fog-enabling computing systems. These systems are based on the traditional client-server-enabled infrastructure or rely on a third-party environment. It poses a serious issue throughout the process of deliverance [36]. For instance, the existing working hierarchy of outsourced computation includes collecting, processing, executing, preserving, and sharing remote sensing data via wireless sensor networks. This unsecure manner increases the rate of integrity, confidentiality, and data redundancy-related problems. In order to protect data from malicious attacks, a modular architecture of blockchain DLT is used. It provides a decentralized platform where data is processed in terms of capturing, scheduling, organizing, managing, optimizing, preserving, and sharing without affecting unknown adversaries. According to this scenario, the current infrastructure of business intelligence is shifting from centralized outsourcing to the newly proposed homomorphic blockchain-based protected outsourcing architecture with cloud and fog computing.

#### 5.1.3 Distributed PSO and ANN for optimal delivery and classification

Various ML algorithms are used to solve complex real-world and combinatorial problems. However, the most successful Artificial Neural Network (ANN) is initiated from scratch, where it takes background knowledge of the problem and then starts considering it [35,37]. Undoubtedly, the overall execution of ANN consumes significant computational energy, time, and bandwidth-related resources. In this scenario, the integration of PSO with ANN resolves several issues, such as being capable of fast convergence when compared with different evolutionary algorithms for the sake of automating meaningful search of ANN architectures for classifications. A secure encoding channel and a velocity operator are required for a system capable of allowing optimization facilities and limiting computational power with the collaboration of ANN, PSO, and blockchain DLT. The main motive is to propose a good, quick, and secure architectural environment where a system can achieve quality performance.

#### 5.1.4 Interoperable chaining limitations

Throughout the development of blockchain technology, interoperable chaining has been one of the most challenging prospects. It occurs when a single transaction goes from one chain to another, which means remote sensing data is delivered, shared, and exchanged from node to node from chain to chain [37,38]. For instance, a complete process cycle consumes more time in executions, and most importantly, it uses high computational bandwidth. To lightweight such a scenario, there is a need to provide a protected interconnected node communication facility, which limits the size of blocks for an individual transaction. It can handle smart production activities such as the collection of remote sensing big data, the organization and management of different values, supply-chain transaction schedules, and a secure real-time monitoring ecosystem. However, the traditional legacy of current industrial architecture also needs improvement, including the lifecycle, service delivery protocols, network structure, intercommunication, and interconnectivity policies, for the sake of designing a secure remote sensing data hierarchy.

## 6 Conclusion

This paper discusses the recent limitations faced by technology in the development of a futuristic, secure social media forensics chain of custody. In order to manage these aspects, this paper introduces the role of ambient intelligence in social media investigation. It highlights the prospects of automation in the pervasive environment, which made the digital investigation more efficient in terms of capturing each log that occurred because of the stop-listen-and-store method association, which indicates the uniqueness of this technology compared to other state-of-the-art schemes. After careful investigation, we found that there is no standardized framework or platform available, even though no process hierarchy is presented. In this paper, we propose a novel framework named ‘ASMF’ that aims to integrate ambient intelligence with federated learning over the distributed consortium network environment to protect the channel of data exchange and preservation on social media platforms. In short, the purpose of using such a collaborative approach is to replace third-party verification and validation processes. In the overall procedure of records organization and optimization, Hyperledger Sawtooth plays a significant role in terms of scheduling node-enabled block transactions in social media forensics for the sake of a secure investigation in cyberspace. For this purpose, there is a single chaincode with four different functions designed to handle the request of each transaction: verification, validation, and chain of custody management of social media forensics. Through the experimental results of the proposed ASMF, a number of simulations are performed, such as computational cost consumption, energy efficiency, usage of network bandwidth, storage, automation, distributed management, organization, and optimization. The evaluation matrices show the fluctuation towards betterment is balanced up to 7.39 %, such as in energy efficiency. The automation accuracy increases up to 3.13 %, whereas the cost of computation and storage capability reduces to 5.34 % and 6.21 %, respectively. Finally, this proposed ASMF is considered a perfect candidate for the future because it can replace the current method of making a social media-enabled chain of custody and the process of digital investigations.

## Data availability statement

No data was used for the research described in the article.

## CRediT authorship contribution statement

**Abdullah Ayub Khan:** Writing - review & editing, Writing - original draft, Visualization, Supervision, Project administration, Investigation, Data curation, Conceptualization. **Xuzhuo Zhang:** Writing - original draft, Software, Resources, Methodology. **Fahima Hajjej:** Software, Funding acquisition, Formal analysis. **Jing Yang:** Writing - review & editing, Project administration, Data curation. **Chin Soon Ku:** Writing - review & editing, Resources, Funding acquisition. **Lip Yee Por:** Writing - review & editing, Supervision, Resources, Funding acquisition, Formal analysis.

## Declaration of competing interest

The authors declare that they have no known competing financial interests or personal relationships that could have appeared to influence the work reported in this paper.
